# Predictors of Beta-Hexachlorocyclohexane blood levels among people living close to a chemical plant and an illegal dumping site

**DOI:** 10.1186/s12940-020-0562-7

**Published:** 2020-01-22

**Authors:** S. Narduzzi, F. Fantini, F. Blasetti, P. Rantakokko, H. Kiviranta, F. Forastiere, P. Michelozzi, D. Porta

**Affiliations:** 1 Department of Epidemiology, Lazio Regional Health Service, ASL Roma 1, Rome, Italy; 2 Department of Prevention, Local Health Unit Roma 5, Colleferro, Italy; 3 Department of Health Security, National Institute for Health and Welfare, Kuopio, Finland

**Keywords:** Human exposure, β-Hexachlorocyclohexane, β-HCH, Contamination, POPs, Chemical wastes

## Abstract

**Background:**

Hexachlorocyclohexane is a synthetic chemical with several isomers, including β-Hexachlorocyclohexane (β-HCH). In 2005, a large contamination of crude milk from some bovine farms along the Sacco River (Central Italy) was detected; it was related to the illegal disposal of large quantities of processing waste by a chemical industry of the area. A biomonitoring study, conducted in 2007 on a sample of the residing population, found high values of β-HCH in people living close to the river. These results led to the establishment of a clinical and epidemiological surveillance program on all the exposed population.

The aim of the study was to evaluate the determinants of β-HCH blood levels in people living within 1 Km of the Sacco River, focusing on the role of specific foods, body mass index and risk factors not yet identified.

**Methods:**

The program involved all people living within 1 km of the river. A descriptive analysis of β-HCH blood levels was done in relation to the potential determinants including specific foods. Regression analysis was used to study the association between potential determinants and (natural log) β-HCH haematic concentration. The results were expressed as geometric mean ratios (GMR). To take into account similarities within the families we adjusted for family clustering.

**Results:**

A total of 602 subjects (87.2%) agreed to participate in the surveillance. The β-HCH geometric mean serum concentration was 72 ng/g lipid. The regression analysis showed that being female (GMR: 1.32, 95%CI: 1.14–1.53), elderly (GMR_> 70yy_: 10.04, 95%CI: 6.65–15.15), obese (GMR: 1.63, 95%CI: 1.28–2.08), eating food of local/own production (GMR 1.47, 95%CI: 1.15–1.88) and using water from private wells (GMR_drink_:1.47, 95%CI: 1.00–2.14 and GMR_wash_: 1.48, 95%CI: 1.17–1.87) were associated with higher β-HCH values. There was inverse association with breastfeeding (GMR: 0.64, 95%CI: 0.47–0.86). The focus on specific foods showed that the most important factors were eggs and beef.

**Conclusions:**

The study indicated a greater contamination for older people, and those drinking and washing with water from private wells and consuming locally produced food, especially eggs and beef.

## Background

Hexachlorocyclohexane (HCH) is a synthetic chemical that exists in eight chemical isomers. Technical-grade HCH was used as an insecticide in Italy and all over the world but it has not been produced or used over 20 years. It typically contained 10–15% gamma-HCH (γ-HCH or lindane) as well as the alpha (α), beta (β), delta (δ), and epsilon (ε) forms of HCH. Virtually all the insecticidal properties resided in γ-HCH that has been recently recognized by the International Agency for Research on Cancer (IARC) as carcinogenic to humans: there is sufficient evidence in humans for the carcinogenicity of lindane for non-Hodgkin lymphoma [1]. The relatively high stability of the HCH isomers in the environment and their global use for many years has led to their continued detection in air, soil, surface water, groundwater, and drinking water [2–4]. The general population can be exposed to HCH through the inhalation of ambient air and the consumption of contaminated food and drinking water [5, 6]. Among the isomers of HCH, β isomer (β-HCH), due to its greater stability, its higher ability to accumulate in fatty tissues (10 to 30 times higher than isomer γ) and its slower elimination from the body (5 times lower than other isomers), is present in higher concentrations in tissues and is therefore more frequently measured [7].

The production and use of lindane, and then of β-HCH, has been prohibited since the beginning of this century in several countries, including Italy (Regulation (EC) No 850/2004), and HCHs have been included in the list of the Persistent Organic Pollutants (POPs) at the Stockholm Convention [8].

The studies on the potential determinants of the human organochlorine concentrations found inconsistent results. In some cases an association with food groups is shown [9, 10]. In other cases only factors such as age, BMI, gender, geography are found to be associated [11, 12].

The Sacco Valley is an area located in Central Italy crossed by the Sacco River. The area was characterized by the presence of a large industrial conglomerate, including a chemical plant producing lindane since the 50’s (Additional file 1: Figure S1). The illegal disposal of chemical waste produced by the plant lead to the contamination of the nearby Sacco River and of the soil within the industrial area. As a consequence of the accumulation of the toxic residuals in the subsoil, in 2005 a large contamination of crude milk of some bovine and ovine farms along the Sacco River was detected [13, 14] . A biomonitoring study on a sample of the population living in the Sacco Valley area was conducted in 2007 showing that people living close to the river (within 1 Km) had the highest observed values of β-HCH. High β-HCH haematic concentration was found especially among older people, those using water from private wells to drink, cook or wash and among those eating the locally produced food [15, 16].

In 2009 a Clinical and Epidemiological Surveillance Program (CESP) on the exposed population living within 1 km of the river was established to evaluate individual and population health in relation to their contamination by β-HCH and to provide information to people exposed on their health status and the need of individual health care. Interviews, laboratory tests and health examinations were scheduled every 3 years.

The aim of this study was to evaluate the predictors of β-HCH blood levels in people living within 1 Km of the Sacco River, participating in the last phase of the CESP, carried out between 2013 and 2015. Compared to the results of the previous biomonitoring study (conducted on a sample of the population of all the Sacco Valley in 2007) [15], we examined in depth two aspects that were not yet evaluated, such as the role of specific foods and the association with BMI.

## Methods

### Population under study

The population of the CESP included all people living (or having land properties) within 1 km of the river in the following municipalities: Colleferro, Segni, Gavignano (province of Roma), Anagni, Sgurgola and Morolo (province of Frosinone) (Additional file 1: Figure S1). We did not have age restrictions although blood testing was not done for individuals below 6 years old. The list of eligible people was prepared on the basis of the addresses recorded at the Municipal Office Registries of the aforesaid municipalities*.* The subjects included in the list were invited by the personnel of the Department of Prevention of Colleferro to visit their medical clinic for a face-to-face interview, anthropometric measurements, cardiovascular examinations and laboratory tests. As the contamination presumably started many years ago, we wanted to be inclusive of all the people potentially exposed, but no longer living in the area. So, we asked to all the participants if any member of their family, currently residing elsewhere, was willing to be included in the study. Very few people were added. Participation was voluntary without any incentive, because the population was very concerned about the contamination.

### Questionnaire

A face-to-face interview using a questionnaire was done asking demographic data, characteristics of the living environment (residence history, property of lands and wells), exposure to chemical and physical agents, smoking and alcohol habits, medical history, fertility, occupational history, use of water from private wells, diet. Questions about lifestyles, including diet, were asked for the period before or until 2005, year of the discover of the massive contamination, and after that date. Information on each food were asked about the frequency (time/week or time/months) and about the origin (commercial local or own production). Data on food consumption up to 2005 have been used in the analyses.

### β-HCH assessment

Thirty cc of blood were taken from each person participating in the CESP at the moment of the interview for the assessment of the haematic level of β-HCH. The blood was processed for the separation of serum and stored at the laboratory of the Department of Prevention in Colleferro at − 20 °C, until the shipment to the National Institute for Health and Welfare, Department of Health Security, in Kuopio, Finland, which conducted the measurement of β-HCH. The method used for the analysis has been described previously in detail [17]. In brief, the method consisted of protein precipitation with ethanol, extraction of β-HCH with dichloromethane–hexane, cleanup with multilayer silica column and analysis with Agilent 7000C gas chromatography – triple quadrupole mass spectrometry (GC–MS/MS) instrument. For quantification ^13^C- labelled internal standards of each compound were used. Two blanks and 1 control sample (NIST SRM1958) were included in each batch of samples (*n* = 22). Measured concentration of β-HCH in SRM1958 was 97%of the certified/reference concentrations and the co-efficient of variation (CV-%) from SRM 1958 was < 5.6%. All results of β-HCH were acceptable, i.e. |Z| < 2, and for most results |Z| < 1. Limits of quantification for β-HCH was 15 pg/ml (3 ng/g lipid given 0.5% fat content in serum).

### Statistical analysis

A descriptive analysis was done to evaluate β-HCH concentration in different subgroups of people, according to their sociodemographic and lifestyle’s characteristics. The concentration of β-HCH was log transformed because of its skewed distribution. Geometric means and geometric mean ratios (GMRs), i.e. the exponentiated regression coefficients, exp.(β), were calculated after adjusting for gender and age.

The possible predictors were chosen based on the plausibility of the biological mechanisms and behavioural factors:

- Gender and age (in classes) [11, 12, 15];
- BMI: overweight or obese (> = 25) vs. normal/underweight (< 25) [11, 12];
- Educational level: high or medium (secondary school or higher) vs. low (primary school or no education) [15];
- Food consumption: consumption (up to 2005) of at least one of the following food of local/own production vs. commercial or no consumption: milk, cheese, eggs, fresh or cooked vegetables, fruit, liver meat, chicken, beef, pork, sheep, rabbit [9, 10];
- Drinking, cooking, irrigating, washing with water from private wells [15];
- Breast feeding (if occurred in the last 15 years) [18, 19].

The correlation between the variables considered was examined. The identification of predictors of β-HCH blood concentration in the population was done by using a regression model with backward stepwise^1^ selection. Since the dependent variable, β-HCH, was not normally distributed, it was transformed and expressed on the log scale. Consequently, the resulting measure of association from the linear regression model was the geometric mean ratio. Then, to focus on the role of specific foods, we performed as many linear regressions as the number of variables related to food consumption. At first, we adjusted only for gender and age, afterwards also for the variables that were important predictors from the stepwise regression model. The regression analyses with only one food at a time could not account for the correlation between the different types of foods, therefore a new backward stepwise^2^ regression model was performed considering simultaneously all the local/own production foods. Since there were some similarities of the individual characteristics within the families, all the models were performed taking into account family clustering, that is correcting standard errors with sandwich estimators. Analyses were performed using the software Stata13.1.

### Results

We contacted 690 subjects offering participation in the second phase of the surveillance program. A total of 602 subjects (belonging to 225 families) agreed to participate with a response rate of 87.2%. No information were collected for no responding people. The people who participated in the surveillance consisted for 51% of males and 43% of people over 50 years (Table 1). The blood concentration of β-HCH was between 2.2 and 2540 ng/g lipid, the median concentration was 71 (SD: 95.7) ng/g lipid, the arithmetic mean was 148 (SD: 243) ng/g lipid, while the geometric mean was 72 (GSD: 3.4) ng/g lipid, indicating an asymmetrical distribution of the pollutant (Skewness: 4.9). Only seven people had a β-HCH serum concentration below the limit of quantification.

**Table 1:** β-HCH (ng/g lipid) GM and GMRs by individual characteristics of the population

				Geometric mean				GMR^a^							
		N	%	Mean	95%CI			Crude	95%CI			Adjusted ^b^	95%CI		
Total		602	100.0	72	65	–	79	–	–	–	–	–	–	–	–
Gender	M	310	51.5	68	60	–	78	1.00				1.00			
	F	292	48.5	76	66	–	88	1.12	0.92	–	1.36	1.21	1.03	–	1.43
Age class (years)	0–18	66	11.0	23	18	–	30	1.00				1.00			
	19–29	60	10.0	39	30	–	50	1.70	1.19	-	2.42	1.70	1.19	-	2.43
	30–39	86	14.3	42	33	–	52	1.81	1.31	-	2.51	1.84	1.33	-	2.55
	40–49	133	22.1	66	55	–	79	2.88	2.13	-	3.89	2.86	2.12	-	3.87
	50–59	96	15.9	110	93	–	130	4.79	3.48	-	6.60	4.77	3.47	-	6.56
	60–69	110	18.3	135	109	–	166	5.88	4.31	-	8.02	5.95	4.36	-	8.11
	> = 70	51	8.5	234	180	–	303	10.20	7.03	-	14.81	10.60	7.30	-	15.39
BMI ^c^	Normal/underweight	200	33.2	43	37	–	50	1.00				1.00			
	Overweight	214	35.5	79	67	–	93	1.84	1.47	-	2.30	1.35	1.11	-	1.65
	Obese	188	31.2	110	94	–	130	2.57	2.04	-	3.23	1.72	1.39	-	2.12
Educational level ^d^	Low	153	25.4	117	93	–	147	1.00				1.00			
	Medium	408	67.8	61	55	–	68	0.52	0.42	-	0.65	0.69	0.55	-	0.87
	High	41	6.8	55	41	–	73	0.47	0.31	-	0.70	0.69	0.47	–	1.02
Family size (people)	1	69	11.5	76	58	–	99	1.00				1.00			
	2	126	20.9	103	84	–	127	1.37	0.96	–	1.95	1.26	0.94	–	1.71
	3	66	11.0	71	52	–	96	0.93	0.62	–	1.40	1.32	0.93	–	1.87
	> = 4	341	56.6	62	55	–	71	0.82	0.60	–	1.12	1.32	1.01	-	1.74
Having a relative with high β-HCH levels (> 150 ng/g lipids)	No	329	54.7	50	45	–	56	1.00				1.00			
	Yes	273	45.3	111	96	–	128	2.21	1.83	-	2.65	2.45	2.11	-	2.85
Breast feeding (in the last 15 years, limited to women aged between 19 and 59 yrs)	No	541	89.9	75	68	–	84	1.00				1.00			
	Yes	61	10.1	46	36	–	59	0.61	0.45	-	0.85	0.64	0.47	-	0.88
Eating local/own production food	No	144	23.9	49	41	–	58	1.00				1.00			
	Yes	458	76.1	81	72	–	91	1.65	1.32	-	2.07	1.72	1.43	-	2.08
Washing with water from private wells	No	308	51.2	58	51	–	66	1.00				1.00			
	Yes	294	48.8	89	78	–	103	1.54	1.27	-	1.86	1.71	1.46	-	2.00
Drinking water from private wells	No	545	90.5	69	62	–	76	1.00				1.00			
	Yes	57	9.5	110	83	–	145	1.60	1.15	-	2.22	1.88	1.43	-	2.48
Cooking with water from private wells	No	346	57.5	63	55	–	71	1.00				1.00			
	Yes	256	42.5	86	74	–	100	1.38	1.14	-	1.68	1.56	1.33	-	1.84
Irrigating with water from private wells	No	275	45.7	59	51	–	68	1.00				1.00			
	Yes	327	54.3	85	74	–	97	1.43	1.18	-	1.74	1.59	1.36	-	1.87

^a^GMR: geometric mean ratio

^b^Adjusted by gender and age classes

^c^BMI categorization:

Normal/underweight: < 25 kg/sm

Overweight: 25–29 kg/sm

Obese: > = 30 kg/sm

Obese: > = 30 kg/sm

^d^Educational level categorization:

Low: junior high school or lower

Medium: high school

High: degree or higher

As shown in Table 1 and in Additional file 1: Figure S2, β-HCH serum concentration increased with age, particularly in people with more than 50 years, and became even higher among people over 70 years. β-HCH level was higher among women compared to men, even when taking into account age. The concentration of β-HCH was more than double for those having at least a relative with high β-HCH level (> 150 ng/g lipid) compared to people who did not, indicating a clear family effect confirmed also after adjusting for gender and age. As reported in the literature [18, 19], there was evidence of inverse association between β-HCH and breastfeeding. The contamination seemed to be greater for those who had eaten at least one food of local/own production and for those who had used water from private wells, located in the contaminated area, for drinking, cooking, washing, or irrigating.

The results of the backward stepwise regression (Table 2) showed that, among the potential predictors, the variables that contributed most to explain β-HCH concentrations were gender, body mass index, breastfeeding, consumption of at least one food of local/own production, drinking and washing with water from private wells. The variables related to wells were somewhat correlated (Additional file 1: Figure S3): the highest Spearman’s correlation coefficients were observed between cooking, irrigating and washing (Corr_c-i_: 0.59; Corr_c-w_: 0.87; Corr_w-i_: 0.66) with water from private wells.

**Table 2:** Association of individual habits and characteristics with β-HCH haematic concentration (ng/g lipid)

	N	%	GMR^a^	*p*-value	95%CI		
Gender							
M	310	51.5	1.00				
F	292	48.5	1.32	0.000	1.14	–	1.53
Age class (years)							
0–18	66	11.0	1.00				
19–29	60	10.0	2.01	0.000	1.37	–	2.94
30–39	86	14.3	2.08	0.000	1.44	–	3.01
40–49	133	22.1	3.32	0.000	2.35	–	4.70
50–59	96	15.9	4.80	0.000	3.30	–	6.97
60–69	110	18.3	5.83	0.000	3.87	–	8.76
> =70	51	8.5	10.0	0.000	6.65	–	15.1
BMI^b^							
Normal/underweight	200	33.2	1.00				
Overweight	214	35.5	1.29	0.019	1.04	–	1.61
Obese	188	31.2	1.63	0.000	1.28	–	2.08
Breast feeding (if occurred in the last 15 years, limited to women aged between 19 and 59 yrs)							
No	541	89.9	1.00				
Yes	61	10.1	0.64	0.004	0.47	–	0.86
Eating local/own production food							
No	144	23.9	1.00				
Yes	458	76.1	1.47	0.002	1.15	–	1.88
Drinking water from private wells							
No	545	90.5	1.00				
Yes	57	9.5	1.47	0.048	1.00	–	2.14
Washing with water from private wells							
No	308	51.2	1.00				
Yes	294	48.8	1.48	0.001	1.17	–	1.87

^a^*GMR* Geometric mean ratio

^b^BMI categorization:

Normal/underweight: < 25 kg/sm

Overweight: 25–29 kg/sm

Obese: > = 30 kg/sm

Standard errors were corrected to account for correlation between subjects nested in families (sandwich estimators)

Table 3 reports the results of the analyses on the role of single foods of local/own production. In particular, foods of local/own production that turned out to be a risk factor were: cheese, eggs, chicken meat, beef, pork, sheep, fresh and cooked vegetables. As expected, eating local or own production fresh vegetables was correlated with consuming local or own production cooked vegetables (Spearman’s correlation: 0.85) (Additional file 1: Figure S3), similarly eating eggs was correlated with eating chicken meat (Spearman’s correlation: 0.73), as a consequence their role in rising β-HCH concentrations in blood may have been overestimated.

**Table 3:** β-HCH (ng/g lipid) GM and GMR from distinct linear regressions, by local/own production food consumed

Food consumption		N	%	Geometric mean	95%CI			GMR^a^	*p*-value	95%CI			GMR^b^	*p*-value	95%CI		
Milk	No	548	91.0	69	62	-	76	1.00					1.00				
	Yes	54	9.0	107	75	-	152	1.43	0.031	1.03	**-**	1.99	1.30	0.130	0.93	-	1.82
Cheese	No	451	74.9	65	58	-	73	1.00					1.00				
	Yes	151	25.1	95	79	-	115	1.39	0.008	1.09	**-**	1.77	1.31	0.021	1.04	**-**	1.65
Eggs	No	205	34.1	48	42	-	56	1.00					1.00				
	Yes	397	65.9	88	78	-	99	1.72	0.000	1.38	**-**	2.14	1.45	0.001	1.16	**-**	1.82
Chicken meat	No	253	42.0	55	48	-	63	1.00					1.00				
	Yes	349	58.0	87	76	-	99	1.63	0.000	1.31	**-**	2.01	1.36	0.005	1.10	**-**	1.68
Beef	No	408	67.8	63	56	-	71	1.00					1.00				
	Yes	194	32.2	93	79	-	111	1.67	0.000	1.31	**-**	2.14	1.44	0.002	1.14	**-**	1.82
Pork meat	No	447	74.3	65	58	-	72	1.00					1.00				
	Yes	155	25.7	97	80	-	117	1.62	0.000	1.27	**-**	2.07	1.40	0.004	1.11	**-**	1.77
Sheep meat	No	445	73.9	63	56	-	70	1.00					1.00				
	Yes	157	26.1	106	87	-	129	1.55	0.001	1.21	**-**	1.99	1.39	0.006	1.10	**-**	1.76
Rabbit meat	No	413	68.6	68	60	-	76	1.00					1.00				
	Yes	189	31.4	82	68	-	98	1.12	0.367	0.88	-	1.43	1.01	0.931	0.80	-	1.28
Liver meat	No	559	92.9	69	62	-	76	1.00					1.00				
	Yes	43	7.1	123	84	-	180	1.50	0.043	1.01	**-**	2.21	1.40	0.097	0.94	-	2.09
Fresh vegetables	No	223	37.0	55	47	-	63	1.00					1.00				
	Yes	379	63.0	84	74	-	95	1.62	0.000	1.31	**-**	2.01	1.35	0.006	1.09	**-**	1.67
Cooked vegetables	No	244	40.5	52	45	-	60	1.00					1.00				
	Yes	358	59.5	89	79	-	101	1.64	0.000	1.34	-	2.01	1.41	0.001	1.15	-	1.72
Fruit	No	505	83.9	69	62	-	77	1.00					1.00				
	Yes	97	16.1	88	68	-	114	1.18	0.225	0.90	-	1.56	1.02	0.861	0.78	-	1.35

^a^*GMR* Geometric mean ratio adjusted by gender and age classes

^b^*GMR* Geometric mean ratio adjusted by gender, age class, bmi, drinking water from private wells, whashing oneself with water from

Standard errors were corrected to account for correlation between subject nested in families (sandwich estimators)

For this reason, a new backward stepwise regression model was performed using simultaneously all the foods of local/own production. The results (Table 4) confirmed the role of gender, age, BMI, breastfeeding, use of water from private wells for drinking and highlighted the key role of consuming local eggs and local beef as a vehicle of contamination.

**Table 4:** Association of individual characteristics and locally produced foods consumption with β-HCH concentration (ng/g lipid)

	GMR^a^	*p*-value	95%CI		
Gender					
M	1.00				
F	1.33	0.000	1.14	–	1.54
Age (years)	1.03	0.000	1.03	–	1.04
BMI (kg/sm)	1.03	0.003	1.01	–	1.04
Breast feeding (in the last 15 years, limited to women aged between 19 and 59 yrs)					
No	1.00				
Yes	0.60	0.000	0.46	–	0.78
Eating local/own production eggs					
No	1.00				
Yes	1.43	0.001	1.15	–	1.78
Eating local/own production beef					
No	1.00				
Yes	1.36	0.011	1.07	–	1.72
Drinking water from private wells					
No	1.00				
Yes	1.67	0.003	1.19	–	2.34

^a^*GMR* Geometric mean ratio

Standard errors were corrected to account for correlation between subjects nested in families (sandwich estimators)

## Discussion

Since 1945 up to the 1970s, the exposure of the Italian population to HCHs was due to the extensive use of lindane for controlling flies and mosquitoes (particularly malaria vectors). Exposure of the population to β-HCH in Italy has never been systematically characterised and the only available data refer to biomonitoring of groups of the Italian general population: Ingelido et al. [20] analysed β-HCH in serum samples of subjects residing in Rome, Brescia, and Naples, enrolled in 2008–2009, and found a median concentration of 18 ng/g lipid; in 2013, Mrema et al. [21] investigated the levels of β-HCH in the blood of a sample of the general population in northern Italy and found a median concentration of 35 ng/g lipid.

In a study of elderly Swedish women (aged 50–74 years) [22], the mean ß-HCH level was 51 ng/g lipid compared to 61 ng/g lipid in the women of our sample. In a study conducted on a representative sample of the population of Catalonia [23], the geometric mean (in ng/g lipid) of ß-HCH was higher than that found in our population (GM: 83 ng/g lipid) indicating some contamination in that area.

The results of this study, together with the history of the environmental characteristics of the area, suggest that the human contamination of β-HCH in the Sacco Valley began in the far distant past (older people have remarkably higher β-HCH serum concentration), mainly through food chain and daily use of water from private wells. The evidence related to specific foods was strong for eggs and beef.

The food chain is supposed to be the main source of human exposure in this case: vegetables irrigated with contaminated water, animals grazing on contaminated soil and fed with contaminated food and water. A positive correlation of β-HCH blood levels with the consumption of food produced in the area was demonstrated in a previous study [15] and clearly confirmed in the present analysis. One of the main interests of this analysis is the role of single food items produced locally. The analysis gave a specific clue in understanding that people were exposed by eating local meat and eggs. Of course, poor reporting of the past dietary habits could be a major concern both for elderly people and for those who were not used to prepare food by themselves. In the literature, fish consumption is a well known source of β-HCH exposure [24, 25], nevertheless in the present study it was not taken into account because the Sacco River Valley is in the inland part of the Lazio region, far from the coast, and fish consumption is rare.

Use of water from private wells seemed to play an important role, especially drinking and washing with water. Recent surveys performed to assess the pollutant concentration in private wells of the area indicated values lower than the maximum concentrations permissible by Italian law (0.1 μg/l) [15]. The apparent inconsistency with the role of well water use found in this study, could be explained either from inadequacy of the water samples taken from wells (water was sampled only on the top of the well whereas the chemical might tend to stay on the bottom) or from the fact that wells were no longer contaminated.

Another focus of this analysis was the role of BMI in the increase of β-HCH body burden. It is difficult to say if this is due to a greater consumption of the contaminated foods or to a higher quantity of adipose tissue where, according to literature [6, 24, 26–28], β-HCH accumulates. The evidence of this study is that the role of BMI remains strong even in fully adjusted model. There are however complicated dynamics between change in adipose tissue volume and toxicokinetics of POPs, such as β-HCH, suggesting a possible reverse causal pathway. Weight loss is responsible for increasing serum concentration of POPs due to reduction in storage capacity in the adipose tissue compartment, which consequently leads to the release of POPs into blood [29, 30]. By contrast, weight gain leads to decreasing serum concentrations of POPs due to a higher storage capacity in the adipose tissue compartment which limits the circulating toxicant burden. Since the accumulation of β-HCH in adipose tissue can reduce the acute burden on other organs or tissues [29–31], such as blood, the different histories of weight change may play a crucial role, more than BMI by itself.

People who participated in CESP were selected because they lived in or owned lands in the area near the Sacco River, thus, people under study were clustered in families and were not independent one another. Usually people in a family share the same lifestyle, the same dietary habits, the same source of water and, not least, a similar genetic code. For this reason, independence among observations was violated and there was a need to correct the standard errors in our analyses. Sandwich estimators were used to apply the correction since the number of groups (families) was large and the number of subjects inside groups (people) was small.

The study has some limitations. Firstly, most people participating to the surveillance program lived in the area within 1 Km of the Sacco River, therefore almost everybody was likely exposed. Secondly, recall bias was a concern because everyone was asked to answer questions about a distant past. As a consequence, the information from people who should have contributed most to the knowledge of exposure in the past are probably those with the largest bias.

Overall, the present analyses confirmed the role of age, local food and private wells but also indicated some new insights about the specific foods that facilitated the contamination.

## Conclusions

The study indicates that β-HCH contamination of the general population living near the Sacco River that was greater for older people, for those who have been drinking and washing with well water, and that it occurred through the food chain, especially through the consumption of locally produced eggs and beef.

## Supplementary information

**Additional file 1:Figure S1.** Map of the Sacco River Valley, Italy. The river, the municipalities and the industrial plant. **Figure S2.** Box-plot of the β-HCH serum concentrations (ng/g lipid), by age class. **Figure S3.** Correlations among different exposures to water from private wells and consumption of local/own production foods

## Data Availability

The datasets analysed during the current study are not publicly available due to privacy issues but are available from the corresponding author on reasonable request.

## Abbreviations

95%CI: 95% confidence intervals;
BMI: Body mass index
cc: cubic centimetres
CESP: Clinical and Epidemiological Surveillance Program
GM: Geometric mean
GMR: Geometric mean ratio
GSD: Geometric standard deviation
HCH: Hexachlorocyclohexane
IARC: International Agency for Research on Cancer
mL: milliliters
ng/g lipid: nanograms/grams of lipid
POPs: Persistent organic pollutants
β-HCH: Beta-hexachlorocyclohexane
γ-HCH: γ-hexachlorocyclohexane

## Footnotes

1 Backward stepwise selection with 0.20 significance level for removal from the model.

2 Backward stepwise selection (0.05 significance level for removal from the model) using gender, age and BMI (linear), use of water from private wells and all the single foods consumption.
