# Supplementary material for: Predictors of Beta-Hexachlorocyclohexane blood levels among people living close to a chemical plant and an illegal dumping site
Source: Environ Health. 2020 Jan 22;19:9. doi: 10.1186/s12940-020-0562-7 (PMC6977344; doi:10.1186/s12940-020-0562-7)
Supplement: Supplementary file 1 — Additional file 1:Figure S1. Map of the Sacco River Valley, Italy. The river, the municipalities and the industrial plant. Figure S2. Box-plot of the β-HCH serum concentrations (ng/g lipid), by age class. Figure S3. Correlations among different exposures to water from private wells and consumption of local/own production foods [file 12940_2020_562_MOESM1_ESM.docx]

Figure 1 – Map of the Sacco River Valley, Italy. The river, the municipalities and the industrial plant


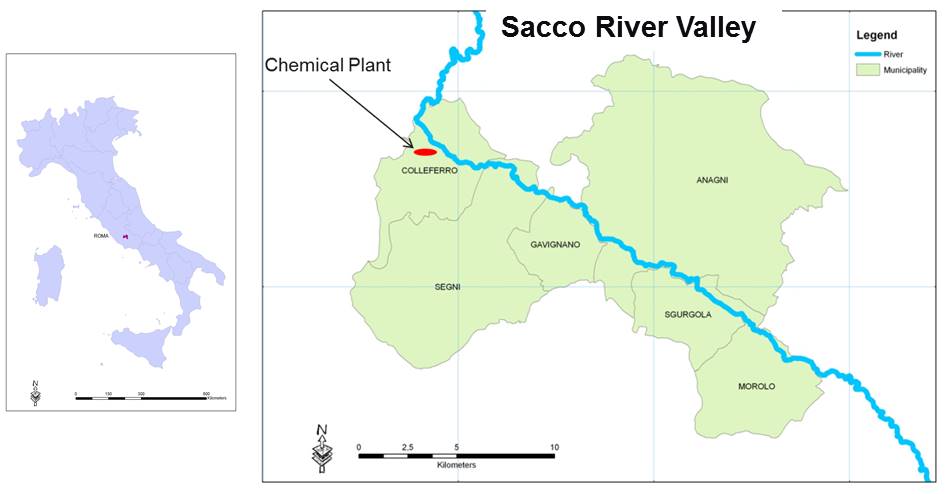


Figure 2 – Box-plot of the β-HCH serum concentrations (ng/g lipid), by age class


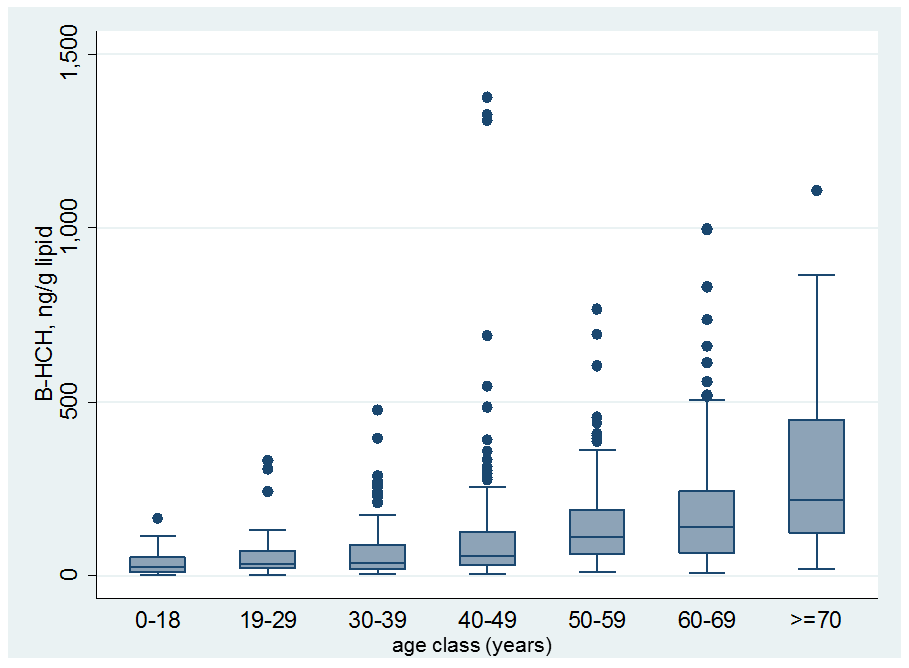


Figure 3 – Correlations among different exposures to water from private wells and consumption of local/own production foods
